# The gamma delta T/NK cell product GADEKILL as a novel immunotherapeutic tool for neuroblastoma patients: role of B7H6 and BTN2A1 in tumor cell killing

**DOI:** 10.3389/fimmu.2026.1755500

**Published:** 2026-01-30

**Authors:** Fabio Morandi, Martina Della Lastra, Fabio Pastorino, Eleonora Ciampi, Maura Faraci, Chiara Brignole, Stefano Giardino, Irma Airoldi

**Affiliations:** 1 UOSD Laboratory of Cell Therapies, IRCCS Istituto Giannina Gaslini, Genova, Italy; 2 UOSD Laboratory of Experimental Therapies in Oncology, IRCCS Istituto Giannina Gaslini, Genova, Italy; 3 Hematopoietic Stem Cell Transplantation Unit, IRCCS Istituto Giannina Gaslini, Genova, Italy

**Keywords:** activating receptors, butyrophilin, immunotherapy, neuroblastoma, NK cells, γδ T lymphocytes

## Abstract

**Background:**

Anti-GD2 monoclonal antibody effectively treats high-risk neuroblastoma (HR-NB) by recruiting NK cells for antibody-dependent cellular cytotoxicity (ADCC). We recently developed a cell product containing mature, cytotoxic γδ T and NK cells (GADEKILL), and its potential use as a novel immunotherapy for HR-NB has been investigated.

**Methods:**

The GADEKILL γδ T and NK cells were analyzed by flow cytometry for the expression of activating and inhibitory receptors and for cytotoxicity against NB, both with and without dinutuximab-β, at a 1:1 effector-to-target ratio. NB cell lines with high and low/absent GD2 expression, as well as patient-derived 3D tumor spheres, all GD2-expressing, were used as targets. Comparative analyses were performed between GADEKILL NK and purified NK cells obtained from the same donor leukapheresis. Furthermore, a panel of NB cell lines was tested for the expression of B7H6 (i.e., NKp30 ligand), Human influenza hemagglutinin-tag (HA-TAG) and calreticulin (i.e., NKp46 ligands), and butyrophilin (BTN)2A1 and BTN3A1/2/3 (i.e., TCRVδ2 ligands), and the impact on GADEKILL cytotoxicity was assessed.

**Results:**

Compared to their purified counterparts, GADEKILL NK cells showed: (i) higher expression of NKp30 and NKp44 and lower expression of CD16 and NKG2D, (ii) greater cytotoxicity (CD107a^+^) against GD2^−^ NB cells, (iii) stronger induction of lysis in low GD2-expressing NB cells and patient-derived 3D tumor spheres, and (iv) comparable ADCC. In addition, both γδ T and NK cells degranulated and consistently induced lysis in a panel of NB cell lines and patient-derived 3D tumor spheres expressing B7H6, calreticulin, HA-TAG, BTN2A1, and BTN3A1/2/3 consistently. Finally, NB cell lysis positively correlated with B7H6 and BTN2A1, and B7H6-blocking experiments revealed a significant decrease in target cell lysis when cells highly expressing B7H6 were used as targets.

**Conclusions:**

Our study demonstrated the potential antineuroblastoma activity of the GADEKILL, supporting its therapeutic use, particularly in the context of relapsed/refractory R/R HR-NB with low GD2 expression.

## 1 Introduction

Neuroblastoma (NB) is the most common extracranial solid tumor in children, with high-risk metastatic disease accounting for 15% of all pediatric oncology deaths. Current first-line treatment for high-risk neuroblastoma (HR-NB) patients includes intensified induction chemotherapy, surgery, high-dose chemotherapy followed by autologous hematopoietic stem cell transplantation (HSCT), radiotherapy, and maintenance therapy with differentiating agents. More recently, immunotherapy with the anti-disialoganglioside (GD2) monoclonal antibody (mAb) dinutuximab-β has been incorporated, significantly improving outcomes for HR-NB patients (1). Despite this intensive multimodal approach, approximately 50% of HR-NB patients still experience tumor relapse and have poor chances of cure (2), with disease-free survival rarely exceeding 10%–15%. Consequently, alternative therapeutic strategies have been explored to improve clinical outcomes. In this context, the combination of chemotherapy and immunotherapy with dinuximab-β has proven effective in inducing a high disease response rate in relapsed patients (3); however, this response tends to decline over time (4, 5). The antineuroblastoma effects of dinutuximab-β, which binds the disialoganglioside GD2 widely expressed on NB cells, are mainly mediated by antibody-dependent cell-mediated cytotoxicity (ADCC), primarily carried out by natural killer (NK) cells (6, 7). One limitation of this immunotherapy is the patient’s immune response, which is often compromised in heavily pretreated patients, as commonly observed in those with relapsed neuroblastoma. Consequently, T-cell-depleted haploidentical HSCT followed by anti-GD2 mAb immunotherapy has been effectively employed as a consolidation strategy in relapsed HR-NB (8). This approach leverages the higher activity of donor-derived immune cells infused with the T-depleted graft and their effective interaction with anti-GD2 antibodies (9), achieving a 5-year-overall survival of more than 50% (10). Furthermore, additional clinical trials have investigated the infusion of donor mature NK cells in combination with dinuximab-β in refractory/relapsed (R/R) HR-NB (11, 12), demonstrating the safety of this approach and promising efficacy. Significant improvements have also been recently reported using autologous GD2-targeting CAR T cells (10, 13, 14). Locatelli et al. (14) documented that GD2-CART01 cells persist in patients for more than 1 year and can induce sustained remission, with a remarkable 5-year event-free survival of 53%, showing better outcomes when administered early in the course of the disease and in patients with low disease burden. Furthermore, Quintarelli et al. (15) explored the use of allogeneic GD2-targeting CAR T cells generated from a fully human leukocyte antigen (HLA)-matched or a haploidentical donor and reported promising results. However, NB with a low to negative GD2 expression—a feature potentially secondary to immunotherapy (16, 17)—appears unresponsive to anti-GD2-targeting CAR T cells, representing a subgroup with further reduced chances of cure.

In this context, we have investigated the potential use of a novel good manufacturing practice (GMP) cell product, named GADEKILL, as an immunotherapeutic tool for R/R HR-NB. We recently reported (Morandi et al. in press) that GADEKILL is composed of mature γδ T lymphocytes, mainly of the Vδ2 subset, and NK cells with cytotoxic potential, which maintain their phenotypic features and functionality after cryopreservation. Such a cell product could potentially be used as a universal off-the-shelf therapy due to the intrinsic characteristics of γδ T lymphocytes and NK cells, and their killing ability mediated by HLA-unrestricted recognition of target cells, thereby avoiding graft-versus-host disease (GvHD) upon infusion into patients (18–20). Indeed, both cell types, which share innate and adaptive-like features (21–27), express CD16 and kill tumor cells through a wide panel of receptors and ligands, including NK group (NKG)2D, NK protein (NKp)30, NKp44, NKp46, and DNAX accessory molecule-1 (DNAM-1), preventing immunological evasion driven by selective antigen loss (28–35).Furthermore, studies from several groups have identified a unique role for members of the butyrophilin (BTN) protein family and have highlighted the crucial roles of BTN2A1 and BTN3A1 in the activation of human γδ T cells by microbial or endogenous phosphoantigens overexpressed by tumor cells (36–47).

Importantly, the enrichment of phosphoantigens in tumors or infected cells is detected by Vγ9Vδ2 T cells, triggering their proliferation and the execution of effector functions required to eliminate these cells (48–50). BTN family members are expressed in various solid tumors, including breast, ovarian, gastric, colon, and pancreatic ductal adenocarcinomas (44). In the context of acute myeloid leukemia, Le Floch et al. (51) recently reported that decreased BTN3A expression on the surface of blasts was associated with poor survival. However, no data are currently available regarding BTN expression in NB.

With this background, we investigated the antitumor potential of GADEKILL against NB by examining the expression patterns of activating receptors and immune-checkpoint molecules on γδ T lymphocytes and NK cells, their degranulation capacity and cytotoxicity toward NB cell lines and patient-derived 3D tumor spheres, and the expression of ligands (i.e., B7H6, calreticulin, and HA-TAG) and BTN on NB cells. Finally, the anti-NB activity of the cell product was compared with that of purified NK cells obtained from the same leukapheresis.

## 2 Materials and methods

### 2.1 Cell production and purification of NK cells

Mononuclear cells (MNC) were obtained using a Ficoll–Hypaque gradient starting from seven leukaphereses of healthy donors, following written informed consent. Four preparations were produced in the laboratory following a previously reported procedure (52), and three were produced in a GMP facility. The GMP process started from three leukaphereses (LA) collected with the Spectra-Optia system (Terumo BCT, Lakewood CO U.S.A.) from healthy donors after informed consent, in accordance with the Declaration of Helsinki (CER Liguria: 593/2021-DB ID 11884). The processed volume ranged from 70 to 103 ml. MNC separated by Ficoll–Paque gradient (Cytiva, Chicago, MA, USA) and CliniMACS Prodigy (Miltenyi), were seeded (5 × 10^6^/ml) in gas-permeable differentiation bags (Miltenyi, Bergisch Gladbach, Germany) and stimulated with zoledronic acid (ZOL, 25 μM; Tillomed by Clinigen, London, United kingdom) in TexMACS medium (Miltenyi), supplemented with 5% human AB-decalcified serum and 1,000 U/ml interleukin (IL)-2 (Proleukin by Novartis, Basilea, Switzerland) for 4 days at 37 °C and 5% CO_2_. The concentrations of IL-2 and ZOL were selected based on previous results (52). Thereafter, cells were transferred to a ZOL-free culture medium (2 × 10^6^/ml) for an additional 10 days, with medium replaced every 3–4 days. At the end of culture, αβ T cells were depleted by CliniMACS Prodigy and T-cell receptor (TCR)αβ biotin and antibiotin reagents (all from Miltenyi).

The four batches produced in the laboratory were obtained using the same culture timing, methodological steps, and clinical-grade reagents as those used in the GMP facility, with the only differences being that MNC were separated using Ficoll–Paque gradient in 50 ml (Falcon, Waltham, US-MA) tubes, and αβ T-cell depletion was performed via immune-magnetic bead manipulation using LD columns (Miltenyi).

To confirm the composition of the final cell product, flow cytometric analysis was performed using a wide panel of fluorochrome-conjugated mAb in different combinations, including CD45, CD3, anti-TCRαβ, anti-TCRγδ, TCRVδ1, TCRVδ2, CD56, CD14, and CD19 (all from Miltenyi). Cells were run on a Gallios^®^ flow cytometer (Beckman Coulter, Brea, CA, USA), acquiring at least 5 × 10^4^ events, and data were analyzed using Kaluza^®^ analysis software (Beckman Coulter). Only pure preparations (> 98% γδT and NK cells) were used. The GMP cell product is hereafter referred to as GADEKILL.The characteristics of NK cells present in the GADEKILL were compared with those of purified NK cells, i.e., which were obtained using immune-magnetic beads with CD3 depletion followed by CD56 enrichment (Miltenyi), starting from the same original samples used for the cell product. Both the cell product and purified NK cells were frozen prior to functional studies. The GADEKILL batches used throughout the study have been cryopreserved for 20–22 months.

### 2.2 Flow cytometry

Flow cytometric studies and analyses followed the MiFlowCyte guidelines (53) and were assessed using the Beckman Coulter Gallios^®^ flow cytometer 1.2. Kaluza software 2.0. was used for data analysis. Cells were stained using fluorochrome-conjugated mAb specific for selected markers, and as a negative control, an isotype mAb of irrelevant specificity conjugated with the same fluorochrome, at the concentration reported in the manufacturer’s data sheets. Cells stained with a combination of fluorochrome-conjugated mAb were analyzed using computed compensation. All the mAb used bind surface molecules, with the exception of 7-amino actinomycin D (7-AAD), a DNA-intercalating dye that is excluded from intact and viable cells but penetrates cells when membrane integrity is compromised, thereby identifying apoptotic or necrotic cells. Percentage analysis was used to determine the expression in nonhomogeneous populations, whereas mean relative fluorescence intensity (MRFI) was used for homogeneous populations, such as NB cell lines. MRFI was calculated as the ratio between the mean fluorescence intensity obtained with the specific mAb and that obtained with an irrelevant isotype control. The gating strategies for the analysis of activating/inhibitory receptors on NK/γδ T cells, as well as for cytotoxicity, are reported in **Supplementary Figure S1**.

### 2.3 Activating and inhibitory receptor analysis

The expression of activating and inhibitory receptors involved in γδ T and NK cell cytotoxicity was assessed by flow cytometry. γδ T and NK cells present in the cell product (identified as CD3^+^ and CD3^−^CD56^+^, respectively) as well as purified NK cells were analyzed using different combinations of the following fluorochrome-conjugated mAb: Fluorescein isothiocyanate (FITC)-NKG2A (clone REA110, Cat. No. 130-113-587), PC7-NKG2C (REA202, Cat. No. 130-120-449), PE-NKG2D (1D11, Cat. No. 557940), Allophycocyanin (APC)-NKp30 (REA823, Cat. No. 130-112-431), PC7-NKp44 (REA1163, Cat. No. 130-120-359), PE-NKp46 (REA808, Cat. No. 130-120-359), CD57 (T1303, Cat. No. 130-122-937), FITC-DNAM-1 (REA1040, Cat. No. 130-117-488) (Miltenyi), and PerCP-Cyanine5.5 (PC5)-CD16 (Cat. No. 407763, Beckman Coulter). Expression of immune checkpoint molecules potentially involved in γδ T and NK cell exhaustion was evaluated using PE-PD1 (clone NAT105, Cat. No. 567617, BD Biosciences, Paramus, NJ, USA), PC5-T cell immunoglobulin and mucin domain containing-3 (TIM-3) (clone 7D3, Cat. No. 567123, BD Biosciences, Paramus, NJ, USA), and APC-T cell immunoreceptor with Ig and ITIM domains (TIGIT) (REA1004, Cat. No. 130-116-815, Miltenyi) mAb. The gating strategy is shown in **Supplementary Figure S1A**.

### 2.4 Expression of γδ T and NK cell ligands on NB cells and B7H6 blocking

A panel of NB cell lines, representing the broad heterogeneity of GD2 expression, and patient-derived 3D tumor spheres (*n* = 4) were analyzed for BTN and Natural cytotoxicity receptors (NCR) ligand expression. Specifically, nine NB cell lines (HTLA-230, IMR-32, LAN-1, LAN-5, GI-ME-N, GI-CA-N, SH-SY-5Y, SK-N-SH, and SK-N-AS) were maintained in culture using DMEM high glucose supplemented with 10% FBS, MEM nonessential amino acid solution, l-glutamine, penicillin, and streptomycin. Patient-derived tumor spheres, characterized by high GD2 expression, were obtained from biological samples of NB patients stored in the repository of the PeRsonalizEd MEdicine (PREME) program. They were processed as described in Capasso et al. (54) and used after dissociation into a single-cell suspension using Accutase.

Patient-derived 3D tumor spheres and NB cell lines were tested for BTN expression using the Alexafluor 647-BTN3A1/2/3 (polyclonal Ab No. FAB7136R, Biotechne, Minneapolis, MN, USA) and PE-BTN2A1 mAb (No. BS9463218, Biotechne Minneapolis, MN, USA). Expression of NCR ligands, including calreticulin and HA-TAG (NKp46 ligand) and B7H6 (NKp30 ligand), was assessed using the following fluorochrome-conjugated mAb: APC–calreticulin (clone 681233, No. IC38981R, R&D System, Minneapolis, MN, USA), FITC HA-TAG (clone not available, No. A01621, GenScript, Piscataway, NJ, USA), and PE-B7H6 (clone jam1ed, No. 12652642, eBiosciences, San Diego, CA, USA). Finally, the B7H6 expression was evaluated in the human leukemia cell lines K562, 697, and NALM-6. At least 5 × 104 events were acquired on a Gallios^®^ flow cytometer.

### 2.5 Cytotoxic activities against NB cells: direct activity and ADCC

The key mechanism underlying the *in vivo* antitumor activity of γδ T and NK cells is related to their cytotoxic abilities, mediated by the release of cytotoxic granules and the lysis of target cells. Cytotoxicity was therefore evaluated using a degranulation assay (CD107a surface expression on effector cells) by coculturing 10^5^ effectors with 10^5^ targets in the presence of 3 µl anti-CD107a (Miltenyi) in 96 V-bottom well plates for 4 h at 37°C in 5% CO_2_. Targets included the following NB samples: (i) HTLA-230 NB cell line, highly expressing GD2 (MRFI: > 600); (ii) SK-N-AS NB cell line, not expressing GD2 (MRFI: < 1.5); and (iii) patient-derived 3D tumor spheres, all expressing GD2 (MRFI: 30.3 to 44.9). Effectors consisted of the GADEKILL and the corresponding purified NK cells. GD2 expression was evaluated by flow cytometry using PE-conjugated anti-GD2 mouse IgG2a mAb (No. 14G2a, BioLegend, San Diego, CA, USA) and dinutuximab-β (Qarziba^®^, Recordati SPA, MIlano, Italy), followed by PE-conjugated antimouse IgG1 mAb (Beckman Coulter). Qarziba^®^ was obtained as a spare aliquot after therapeutic use.

Analyses were performed by flow cytometry, and degranulating effector cells were identified as CD45^+^CD3^+^CD107a^+^ (γδ T) and CD45^+^CD3^−^CD107a^+^ (NK) cells within the physical lymphocyte gate. NB cells were discriminated on their physical properties and negative CD45 expression, and NB cell lysis was evaluated by 7-AAD staining (Miltenyi). Target cell lysis was defined as 7-AAD-positive cells within the CD45^−^ gated cells. Negative controls included effectors and targets alone, and the myeloid leukemia K562 cell line was used as a positive control. The gating strategy is shown in **Supplementary Figure S1B**.

Studies on ADCC driven by both γδ T and NK cells present in our cell product, or by purified NK cells, were performed using 3 µg/ml dinutuximab-β in a degranulation assay.

### 2.6 Correlation of NCR ligand and BTN expression on NB cells and target cell lysis

Additional studies were conducted to determine whether the killing activity of GADEKILL correlated with the expression of B7H6, calreticulin, HA-TAG, BTN2A1, and BTN3A1/2/3. To this end, cytotoxicity was analyzed using the nine NB cell lines described above and two patient-derived 3D tumor spheres as targets, with three cryopreserved cell products as effectors. Correlations between cell lysis and the expression of B7H6, HA-TAG, BTN2A1, or BTN3A1/2/3 on target cells were assessed by Spearman’s rank correlation analysis with a 90% confidence interval using GraphPad Prism 10.5 software.

Additional experiments were conducted to assess whether B7H6 contributes to GADEKILL-mediated cytotoxicity. To this end, cytotoxicity was evaluated as described above by adding 10 μg/ml of a blocking B7H6 mAb (R&D System) to the target cells 30 min before the coculture with effector cells. The targets included two human cell lines with high B7H6 expression (GI-CA-N NB and K562 leukemia cells) and two with low B7H6 expression (IMR32 NB and NALM-6 leukemia cells).

### 2.7 Statistical analyses

Statistical analyses were performed using Prism software (GraphPad Prism 10.5 software, GraphPad Inc., Boston, USA). The Mann–Whitney *U* test was applied because the data distribution was not Gaussian, as assessed by the D’Agostino and Pearson normality tests. Correlations were analyzed using the Pearson test. All statistical tests were one-tailed, and a *p*-value below 0.05 was considered statistically significant.

## 3 Results

### 3.1 Expression of activating and inhibitory receptors in γδ T cells present in the GADEKILL

The GADEKILL exhibited consistent cell viability (> 97%), as assessed by 7-AAD staining and flow cytometric analysis, and was composed of NK cells (> 60%) and γδ T cells, which were mainly of the Vδ2 subtype (consistent > 90%). In all batches, γδ T lymphocytes were predominantly of the effector memory phenotype (> 70%), with smaller subsets of naïve, central memory, and terminally differentiated cells (data not shown). Monocytes were virtually absent, whereas B lymphocytes and αβ T cells were present at very low frequencies (< 2.5% and 0.3%, respectively). This information has been previously reported (Morandi et al. in press).

**Figure 1:**
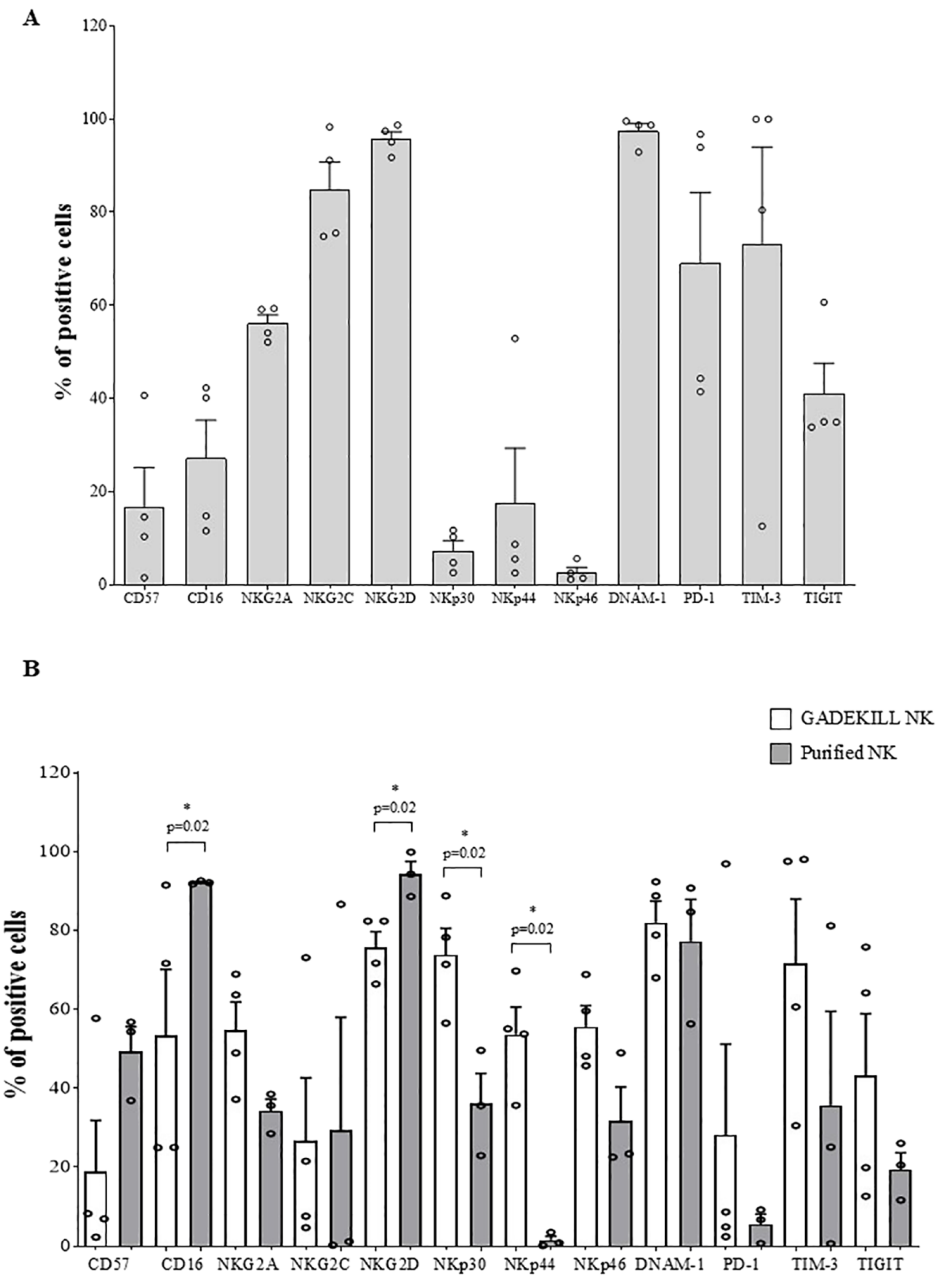
Expression of receptor involved in cytotoxicity of γδ T and NK cells present in the GADEKILL and in purified NK cells from the same leukapheresis donor. **(A)** Expression of a panel of activating and inhibitory receptors was tested by flow cytometry and analyzed in gated lymphomononuclear cells. γδ T lymphocytes in the GADEKILL (*n* = 4) were identified as CD3^+^ cells. Results are expressed as the percentage of γδ T-expressing cells ± standard error (SE). **(B)** Comparative analysis of activating and inhibitory receptors on NK cells present in GADEKILL (identified as CD3^−^, white bars) and on purified NK cells (black bars) from four leukapheresis donors is reported. Results from flow cytometric analyses are expressed as the mean percentage of NK-expressing cells ± SE, and asterisks correspond to significant differences, evaluated by the Mann–Whitney *U* test.

We first evaluated the expression of a panel of γδ T-cell receptors involved in cytotoxicity by flow cytometry, using four GADEKILL batches produced in a research laboratory. As shown in **Figure 1A**, consistent expression, albeit at varying levels, was observed for the activating receptor CD57 (mean %: 16.8%, range: 1.6% to 40.6%), which is considered a marker of effector memory T lymphocytes with high cytotoxic activity and not necessarily indicative of exhaustion (55). CD16, the FcγRIII receptor mediating ADCC, was present at a mean percentage of 27.2 (range: 11.6% to 42.3%). NKG2A, NKG2C, and NKG2D were also consistently expressed, with mean percentages of 56.2% (from 52.1% to 59.3%), 84.9% (from 74.8% to 98.3%), and 95.7% (from 91.8% to 98.7%), respectively. The NCR tested were detected at mean percentages of 7.6% for NKp30 (from 2.7% to 11.7%), 17.4% (from 2.6% to 52.9%) for NKp44, and 2.7% for NKp46 (from 1.2% to 5.7%), whereas DNAM-1 was expressed at a high percentage (mean %: 97.5%, range: 92.9% to 99.5%). Furthermore, all the inhibitory receptors assessed were found to be expressed on γδ T lymphocytes present in the cell product (mean % of Programmed cell death protein 1 (PD-1): 69.1%, range: 41.5% to 96.7%; TIM-3: 73.3%, range: 12.6% to 100%; TIGIT: 41.1%, range: 33.9% to 60.7%). Of note, PD-1 and TIGIT have recently been reported to exert dual functions in γδ T cells, as they may be considered hallmarks of activation and effector function rather than exclusively markers of functional exhaustion (28, 56).

### 3.2 Activating and inhibitory receptors in GADEKILL NK compared to purified NK cells

Flow cytometric analyses revealed significant differences (*p* = 0.02) in the expression of CD16, NKG2D, NKp30, and NKp44 in GADEKILL NK (white bars) compared with NK cells (black bars) purified by immune-magnetic beads from the same starting material (**Figure 1B**). In particular, GADEKILL NK cells exhibited higher expression of NKp30 and NKp44 (mean %: 73.8% and 53.6% versus 43.3% and 1.53%, respectively), which was associated with lower expression of CD16 and NKG2D (mean %: 62.8% and 78.8% versus 92.2% and 94.2%). The other activating receptors, NKG2C, NKp46, and DNAM-1, were expressed without significant differences (mean %: 26.7% versus 29.4%; 55.6% versus 31.7%; and 82% versus 77.3%, respectively). Finally, GADEKILL NK cells and purified NK cells showed comparable levels of the inhibitory NKG2A (mean %: 54.7%, range: 37.2% to 68.9% versus mean %: 34.3%, range: 28.5% to 38.5%), as well as the immune-checkpoint molecules PD-1 (mean %: 28.2%, range: 2.4% to 96.9% versus mean %: 5.6, range: 0.7 to 9.2), TIM-3 (mean %: 71.7%, range: 30.6% to 98.1% versus mean %: 35.7%, range: 0.7% to 81.2%), and TIGIT (mean %: 43.1%, range: 12.7% to 75.8% versus mean %: 19.5%, range 11.7% to 26.1%).

Afterwards, we have investigated whether the different expression of the receptors observed could impact the cytotoxic abilities against NB cells.

### 3.3 NK cell cytotoxicity and ADCC against NB cells: GADEKILL versus purified NK cells

The direct cytotoxicity, as well as the ADCC exerted by NK cells in the four GADEKILL batches produced in the laboratory, compared with purified NK cells, was tested by a degranulation assay using two NB cell lines as targets (i.e., the HTLA-230, which highly expresses GD2, and the SK-N-AS, which is negative for GD2) and three 3D tumor spheres with high levels of GD2. The human myeloid leukemia cell line K562 was used as a positive control of NK cell cytotoxicity.

**Figure 2:**
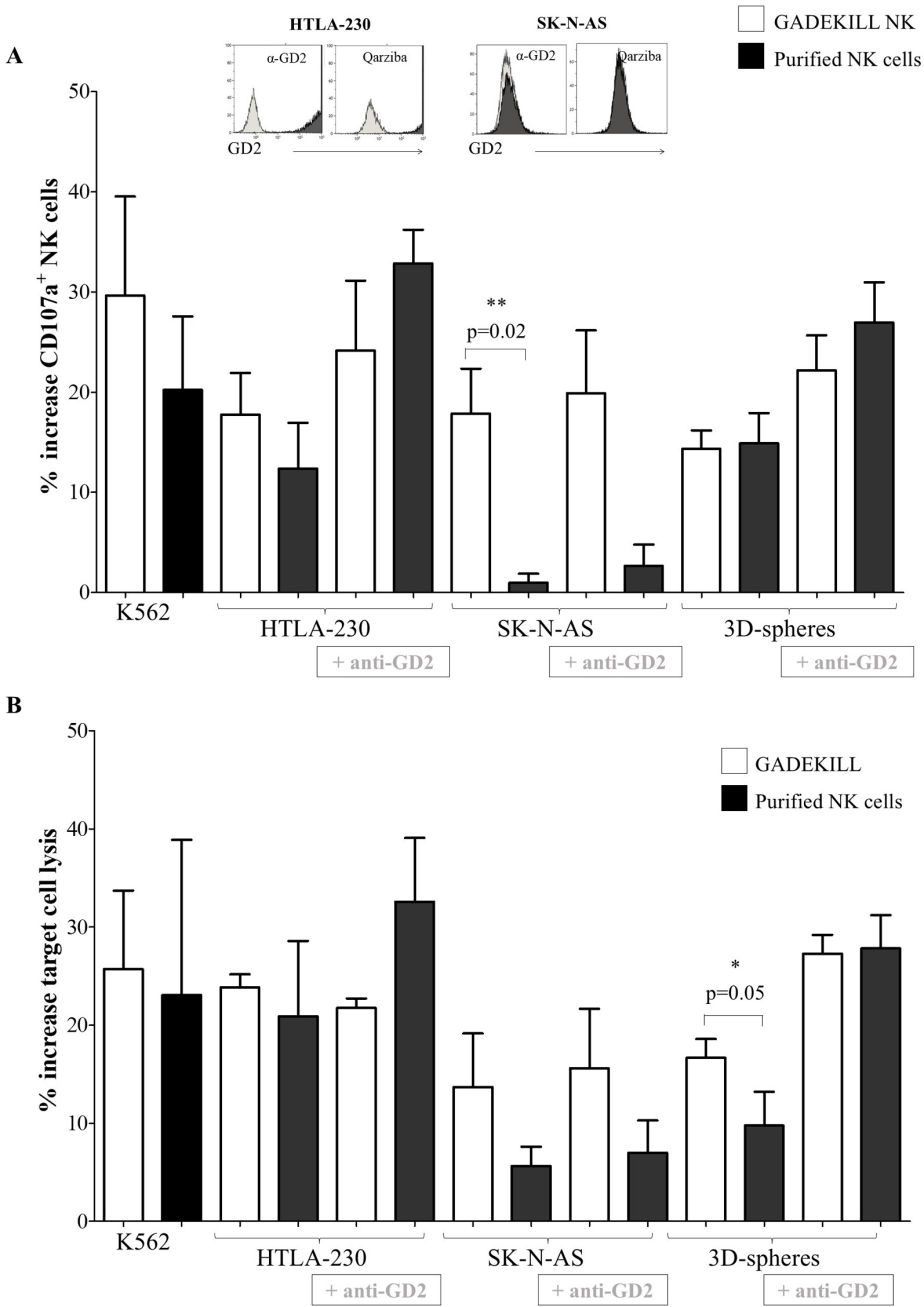
Direct cytotoxicity and ADCC against NB cells. **(A)** Comparative analysis of GADEKILL NK (white bars) and purified NK (black bars) cell degranulation in response to target NB cells, namely HTLA-230, highly expressing GD2 (as shown in the inset, left), SK-N-AS, low expressing GD2 (as shown in the inset, right) and 3D tumor spheres, in the absence or presence of Qarziba (+ anti-GD2), was performed by flow cytometry. GADEKILL NK and purified NK were obtained from the same leukapheresis donor (*n* = 4). Effectors and target cells were cocultured for 4 h at a 1:1 ratio. Results are expressed as the mean percentage increase of CD107a surface expression (mean % of CD107a^+^ effector cells cultured in the presence of target − mean % of CD107a^+^ effector cells cultured in the absence of target). Asterisks indicate statistically significant differences, evaluated by the Mann–Whitney *U* test. The K562 cell line was used as a positive control. **(B)** Comparative analysis of NB cell lysis (identified as 7-AAD^+^ NB cells) induced by GADEKILL (*n* = 4, white bars) or by purified NK cells (*n* = 4, black bars) obtained from the same donor was assessed by flow cytometry. ADCC was tested with the addition of Qarziba (+ anti-GD2) in the coculture system. Target NB cells were identified through the exclusion of CD45^+^ cells and by physical gating. Results are expressed as the mean percentage increase 7-AAD^+^ target cells ± SE (mean % of 7-AAD^+^ NB cells cultured in the presence of effector − mean % of 7-AAD^+^ NB cells cultured alone). An asterisk corresponds to a significant difference evaluated by the Mann–Whitney *U* test.

As reported in **Figure 2A**, NK cells in the GADEKILL (white bars) exhibited higher, although not statistically significant, CD107a surface expression when cocultured with K562 and HTLA-230 cells. Specifically, the mean percentage of CD107a^+^ NK cell product against K562 was 42.9%, ranging from 36.7% to 49.2%, whereas against HTLA-230 cells, the mean percentage of CD107a^+^ purified NK cells was 34.7%, from 24.9% to 47.2%. These values were higher than those observed in purified NK cells (black bars), which showed a mean CD107a^+^ percentage of 33.9% (range: 17.3% to 50.7%) against K562 and 26.6% (range: 4.4% to 45.2%) against HTLA-230 cells. The mean basal expression (i.e., in the absence of target cells) of CD107a, defined as expression in the absence of target cells, was 18.7% in GADEKILL NK cells and 12.1% in purified NK cells. By contrast, a statistically significant difference (*p* = 0.02) was observed when SK-N-AS cells were used as target cells. Under these conditions, CD107a was expressed at a mean level of 34.8% (range: 22.2% to 51.1%) in GADEKILL NK and 11.3% (range: 4.5% to 19.1%) in purified NK cells. Furthermore, no statistically significant differences were observed when 3D tumor spheres were used as targets. Notably, the effector-to-target ratio used in the cytotoxicity studies was highly challenging for the cell product, as it did not account for the relative proportion of NK cells within GADEKILL, which ranged from 64.1% to 78.2% (data not shown). Therefore, the comparable or higher NK cell cytotoxicity observed may indicate greater antitumor activity of GADEKILL NK compared with the purified NK cells.

The data reported in **Figure 2A** are expressed as a percentage increase in CD107a expression, defined as the percentage of CD107a on effector cells in the presence of target *minus* (−) the percentage of CD107a on effector cells in the absence of target cells.

Both GADEKILL NK and purified NK cells increased degranulation when the anti-GD2 mAb dinutuximab-β was present in the coculture with GD2-expressing target cells (**Figure 2A**); no statistically significant differences were observed.

Additional analyses were performed to investigate whether the cell product could induce more efficient target cell lysis, considering that γδ T lymphocytes are also present in the GADEKILL product and may contribute to this activity. The basal level of cell death (i.e., mean % of 7-AAD^+^ cells) for each target was 4.7% in K562, 28.7% in HTLA-230, 21.8% in SK-N-AS, and 30.3% in 3D tumor spheres. As shown in **Figure 2B**, the % of 7-AAD^+^ target cells did not differ significantly between GADEKILL (white bars) and purified NK cells (black bars) for any tested target, with the only exception being 3D tumor spheres (*p* = 0.05, mean % of 7-AAD^+^ target cells: 47.9%, range: 31.6 to 62.6% with GADEKILL *versus* 39.5%, range: 29.3% to 55.5% with purified NK). The mean percentage increase in 3D tumor cell lysis was 16.7% and 9.7%, respectively.

Although the difference was not statistically significant, a trend of higher cell lysis was observed when SK-N-AS was used as the target and the cell product as the effector.

### 3.4 Cytotoxicity of the GADEKILL cell product against a panel of NB cell lines and patient-derived 3D tumor spheres

**Figure 3:**
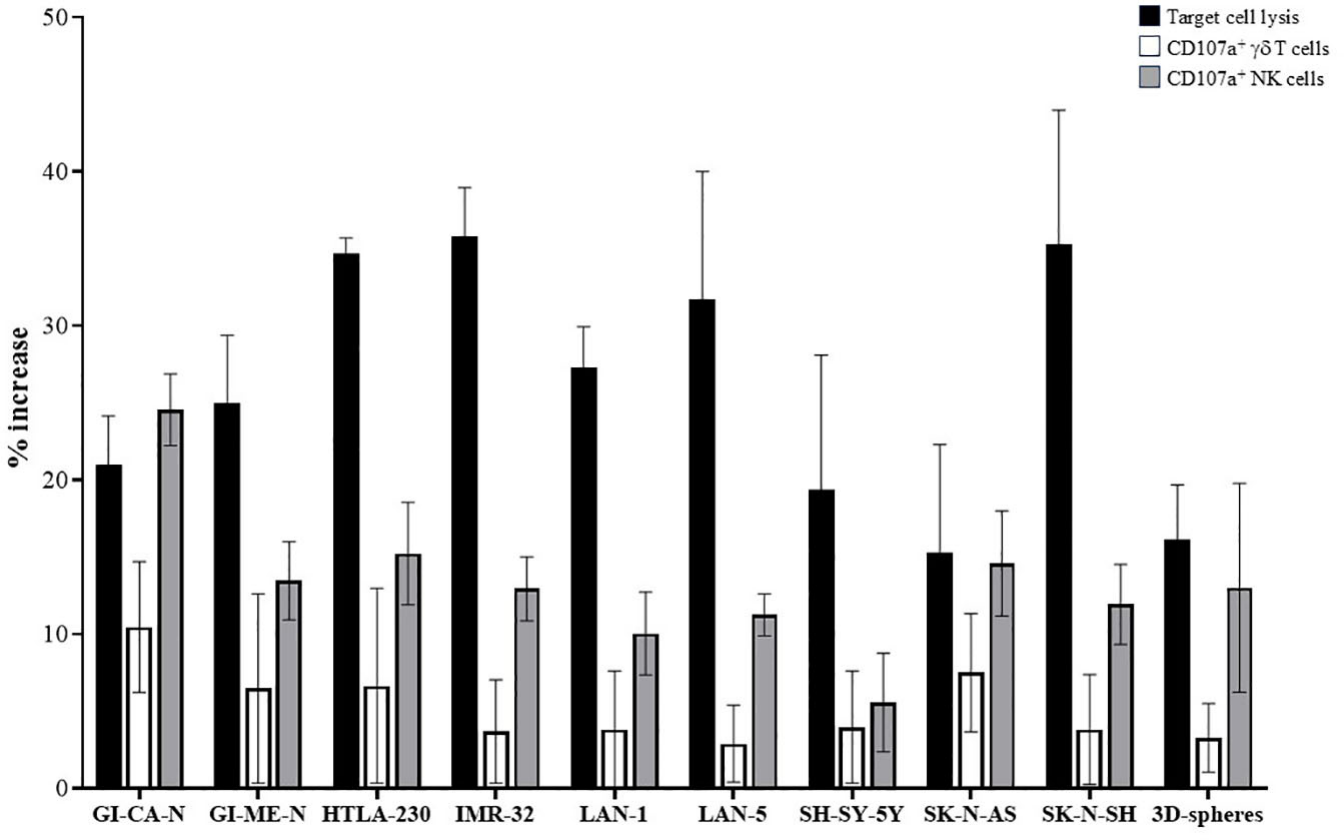
Cytotoxic activity of the GADEKILL against a panel of human NB cell lines and patient-derived 3D tumor spheres. The cytotoxic ability of the GADEKILL against NB cell lines with different n-MYC status and GD2 expression, as well as patient-derived 3D tumor spheres, was assessed by flow cytometry and a degranulation assay at a 1:1 GADEKILL: NB ratio. Three different batches of GADEKILL were used. Cytotoxicity was tested in terms of target cell lysis induction (black bars) and CD107a surface expression on γδ T cells (white bars) and NK cells (grey bars). γδ T lymphocytes and NK cells in the GADEKILL were identified as CD3^+^ and CD3^−^ cells, respectively. Results are expressed as the mean percentage increase of CD107a surface expression (mean % of CD107a^+^ effector cells cultured in the presence of target − mean % of CD107a^+^ effectors in the absence of target) ± SD. Pooled results from three samples of patient-derived 3D tumor spheres are shown.

In order to provide evidence that the cell product may represent an effective immunotherapeutic tool against NB, we extended our cytotoxic studies using a panel of NB cell lines representing broad heterogeneity in GD2 expression. Specifically, SK-N-AS and GI-ME-N expressed low/absent levels of GD2 (MRFI < 1.5), whereas the other cell lines tested showed high GD2 expression (MRFI > 30). Cytotoxicity of the GADEKILL batches produced in a GMP facility or in a research laboratory was evaluated, as described above, in terms of degranulation (CD107a surface expression) of both γδ T and NK cells in the cell product, as well as NB target cell lysis. As mentioned, the 1:1 effector-to-target ratio does not account for the relative proportions of NK and γδ T cells. As shown in **Figure 3** and **Supplementary Figure S2**, the cell product efficiently killed target NB cells, as indicated by the induction of 7-AAD^+^ in CD45^−^ target cells (black bars) and by increased CD107a surface expression on both γδ T (white bars) and NK cells (grey bars). Degranulation in NK cells was consistently higher than in γδ T lymphocytes, regardless of the NB cell line used. Notably, similar results were obtained using patient-derived 3D tumor spheres as targets.

### 3.5 NCR ligands and BTN in NB cell lines and blocking B7H6 results

**Figure 4:**
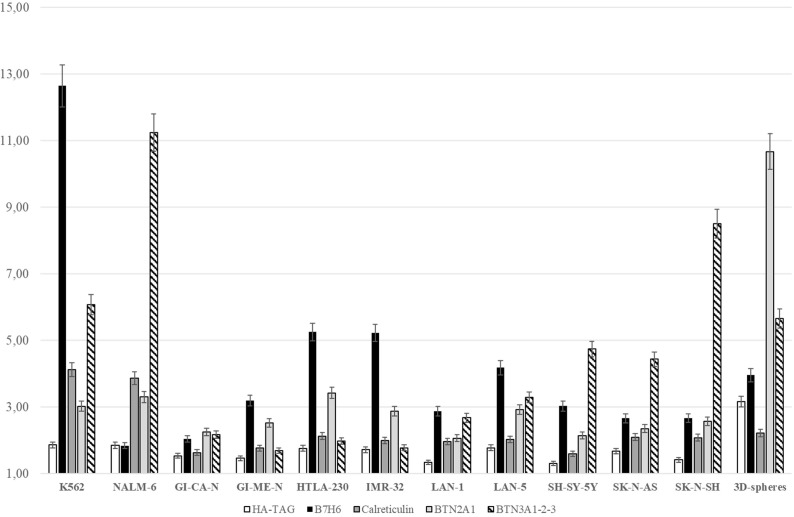
HA-TAG, B7H6, calreticulin, and butyrophilins in human NB cell lines and patient-derived 3D tumor spheres. Flow cytometric analysis was performed to assess the expression of the NKp46 ligands HA-TAG (white bars) and calreticulin (dark gray bars), the NKp40 ligand B7H6 (black bars), BTN2A1 (light grey bars), and BTN3A1/2/3. Results from two independent experiments for each individual NB cell line, as well as pooled data from three different patient-derived 3D tumor spheres, are reported as mRFI.

**Figure 5:**
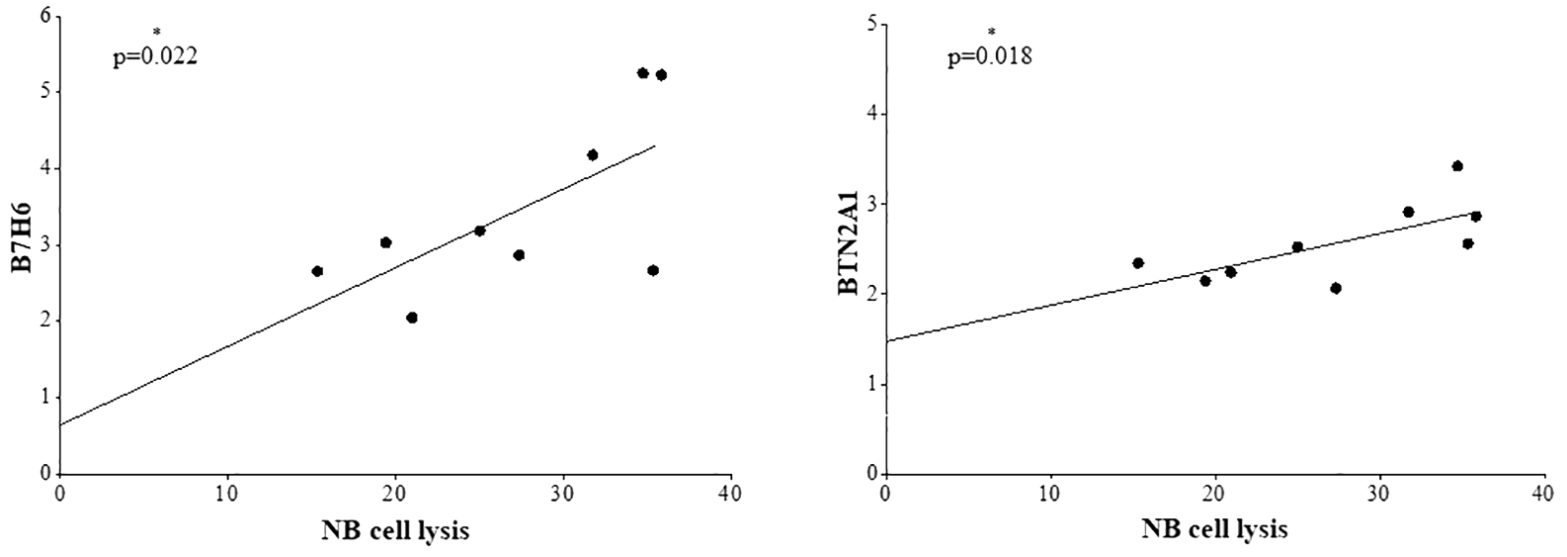
Correlation analyses of B7H6 and BTN2A1 expression with NB cell lysis. Analyses were performed by correlating the NB cell lysis (7-AAD^+^ cells) with the surface expression of B7H6 (left) and BTN2A1 (right) on NB cells. Pearson correlation analysis with a 90% confidence interval was conducted using GraphPad Prism version 10.5 software. The data passed the KS, D’Agostino and Pearson, and Shapiro–Wilk normality tests. The goodness of fit was 0.459 (*R*-squared) for B7H6 and 0.458 (*R*^2^) for BTN2A1. Asterisks indicate significant correlations.

Due to the variable cytotoxicity observed against NB cells, we next analyzed the expression of the NCR ligands B7H6, calreticulin, and HA-TAG, as well as BTNA21 and BTN3A1/2/3, on NB cell lines using flow cytometry. These ligands were selected due to the following considerations: (i) NKp30 and NKp46 are expressed by both γδ T and NK cells, and B7H6 has been reported to have clinical relevance in high-risk neuroblastoma patients (57), (ii) calreticulin and HA-TAG represent NKp46 ligands with unexplored activity in NB, (iii) BTN molecules are known to induce selective γδ T-cell cytotoxicity through interaction with the Vδ2 chain, and the GADEKILL population is composed predominantly of the Vδ2 subset, and (iii) the availability of mAb for phenotypic analysis and blocking experiments. As shown in **Figure 4**, all the ligands analyzed were expressed on NB cell lines, although at different levels, as measured by MRFI. Specifically, HA-TAG (light grey bars) and calreticulin (dark grey bars) were expressed at low intensity (mRFI from 1.3 to 1.8 and from 1.6 to 2.13, respectively), B7H6 (black bars) at variable levels with mRFI from 2.05 in GI-CA-N to 5.25 in HTLA-230, BTN2A1 with intensity ranging from 2.1 to 3.43, and BTN3A1/2/3, with the lowest expression in GI-ME-N and the highest in SK-N-SH cells. Next, we investigated whether the lysis of target cells induced by the cell product could be correlated with the expression of these ligands. As reported in **Figure 5**, B7H6 and BTN2A1 on NB cells positively correlated (*p* = 0.022 and 0.018, respectively) with target cell lysis, whereas calreticulin, HA-TAG, and BTN3A did not (data not shown). Finally, no positive or negative correlation was observed by analyzing the expression of the same ligands to the degranulation of γδ T and NK cells (data not shown).

**Figure 6:**
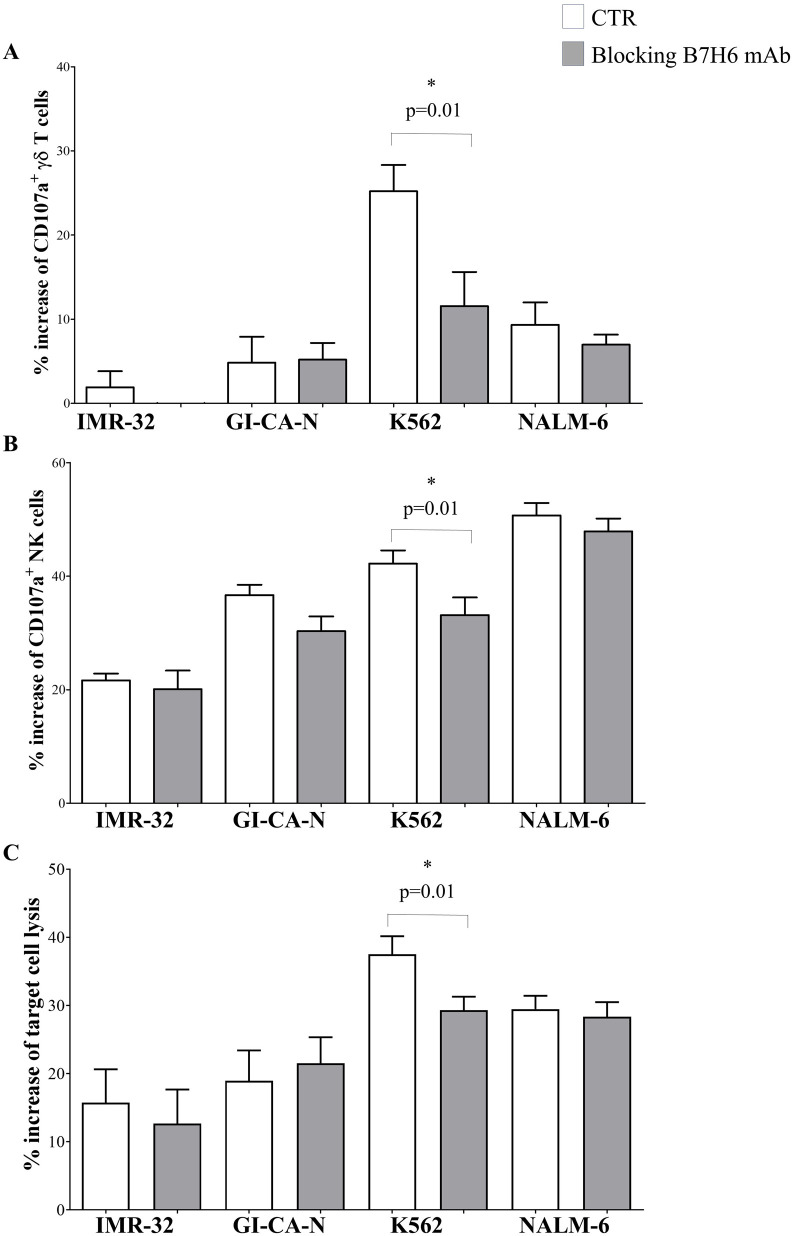
Modulation of GADEKILL cytotoxicity by blocking B7H6. Degranulation of γδ T **(A)** and NK **(B)** cells in the presence of two NB cell lines (IMR-32 and GI-CA-N) and two leukemic cell lines (K562 and NALM-6) was assessed after pretreatment of target cells with a blocking B7H6 mAb for 30 min prior to coculture with effectors. IMR-32 and K562 were selected due to high B7H6 expression, whereas GI-CA-N and Nalm-6 were selected due to low B7H6 expression. Target cells not pretreated with the B7H6 mAb (CTR) are shown in white columns, whereas those with a blocking B7H6 mAb are shown in grey columns. Results are expressed as the mean percentage increase in CD107a surface expression ± SE (mean % of CD107a^+^ effector cells cultured in the presence of target − the mean % of CD107a^+^ effector cells cultured in the absence of target). Three different experiments were performed. Asterisks indicate statistically significant differences. **(C)** The target cell lysis induced by the cell product was analyzed by 7-AAD staining and flow cytometry. IMR-32, GI-CA-N, K562, and NALM-6 were pretreated with a blocking B7H6 mAb for 30 min (grey columns) prior to coculture with effectors. Controls (CTR) consisted of the same target cells without B7H6 mAb pretreatment. Results are expressed as the mean percentage increase in 7-AAD^+^ target cells ± SE (mean % of 7-AAD^+^ NB cells cultured in the presence of effectors − mean % 7-AAD^+^ NB cells cultured alone). Asterisks indicate statistically significant differences, as evaluated by the Mann–Whitney *U* test.

Subsequently, we tested whether the B7H6 blocking affects the GADEKILL cytotoxicity. To this end, a B7H6-blocking mAb was used to treat one NB cell line with high expression of B7H6 (i.e., IMR-32, mRFI: 5.23) and one with low expression (i.e., GI-CA-N, mRFI: 2.05) prior to the cytotoxicity assay. Two additional leukemia cell lines, K562 and NALM-6, were included due to their very high and very low B7H6 expression (12.65 and 1.84, respectively). As shown in **Figure 6**, the B7H6-blocking mAb significantly inhibited the degranulation of γδ T (**Figure 6A**) and NK cells (**Figure 6B**) when cultured with K562, as well as the induced target lysis (**Figure 6C**). Cytotoxicity was not impaired when GI-CA-N and NALM-6 cells were used. However, a trend toward inhibition of γδ T-cell degranulation and target cell lysis was observed with IMR-32, which expresses a lower intensity of B7H6 compared with K562. Taken together, these experiments highlight the importance of B7H6, as well as BTN2A1, in GADEKILL-mediated cytotoxicity against NB cells.

## 4 Discussion

The 5-year survival rate of patients with HR-NB is approximately 50%, despite the use of highly aggressive and life-threatening treatment approaches. Worldwide efforts are ongoing to develop novel and more effective therapies to improve patient outcomes (58, 59). Immunotherapy using anti-GD2 mAb has improved the overall survival of patients with HR-NB to 60% (60), and small case studies have reported encouraging event-free survival rates of up to 75% with the addition of other immunotherapeutic strategies (1, 5, 9, 10, 61–63). However, analyses of patients with relapsed or refractory disease following anti-GD2 mAb therapy have identified cases showing downregulation or complete loss of GD2 expression on NB (16, 17, 64). Very recently, substantial improvements in response rates were reported using consolidation treatments after NB relapse. These approaches included, on the one hand, T-depleted haplo-HSCT as a platform for subsequent mAb-based immunotherapy (10) and, on the other hand, both autologous and allogenic GD2-directed CAR T cells in R/R HR-NB (13–15). Nonetheless, further enhancement of long-term immune responses still appears to be required for both platforms. Moreover, patients with negative or low GD2 expression do not seem to benefit from CAR T-cell approaches and therefore remain without an effective therapeutic option.

In this context, we developed a novel cell product composed of γδ T and NK cells, named GADEKILL, which was recently authorized for therapeutic purposes (Am1/25 by Agenzia Italiana Farmaco [AIFA]). The antitumor effects of this cell product take advantage of the complementary properties of γδ T lymphocytes and NK cells, which kill tumors in an MHC-unrestricted manner and independently of specific tumor antigen expression. Importantly, both cell populations do not induce GvHD in the recipient and are virtually free of toxicity. The novel characteristics of the GADEKILL, compared to other published data (65–70), include the presence of a high proportion of NK cells (> 60%) obtained through a GMP process, which involves (i) the use of decalcified human AB serum and biobags for cell culture, (ii) timing of αβ T-cell depletion and cell expansion, and (iii) the absence of engineered artificial antigen-presenting cells. The high proportion of NK cells in the final cell product was not related to the NK cell content in the starting material, as previously reported by others (66).

We focused on the antitumor potential exerted by the GADEKILL against NB with high and low GD2 expression using a panel of NB cell lines and 3D tumor spheres generated from NB patients. Notably, this preclinical model recapitulates the histological and genomic features of the originating tumors and has been proven to be a valuable asset for translational research (54). We documented that the cell product was able to kill NB target cells directly and through ADCC, and that both γδ T and NK cells degranulated after coculture for 4 h, due to the presence of activating receptors on effector cells and the corresponding ligands on NB cells. The γδ T and NK cells in the GADEKILL exhibited an activating and cytotoxic phenotype, as indicated by the expression of CD16, NKG2D, NKG2C, CD57, DNAM-1, and NCR. NKG2A functions as an inhibitory receptor on NK cells through recognition of the HLA-E molecule (71) but acts as an activating receptor on γδ T cells (72) and was found to be consistently expressed. In addition, both γδ T and NK cells expressed immune-checkpoint molecules commonly associated with suppression and exhaustion, such as TIGIT, PD-1, and TIM-3. However, the expression of these molecules was not linked to exhausted functionality, as supported by the documented cytotoxicity and the proliferative capacity (Morandi et al. in press). This behavior may be related to the observation that these molecules were more highly expressed in γδ T cells, where they may function as activation and effector molecules (73, 74).

Comparative analyses between GADEKILL NK and purified NK cells obtained from the same donor revealed that only a few activating receptors were differently expressed: CD16 and NKG2D were present at higher percentages in purified NK cells, whereas NKp30 and NKp44 were expressed at lower levels. Nonetheless, both GADEKILL NK cells and purified NK cells equally lysed NB target cells in the presence of anti-GD2 mAb, indicating that the differences in CD16 levels do not impact ADCC. In addition, GADEKILL NK cells showed higher degranulation when cocultured with GD-NB cell lines and induced more effective lysis of patient-derived 3D tumor spheres, highlighting the therapeutic potential of this cell product. Notably, the effector-to-target ratio used in our cytotoxicity studies was very challenging for the cell product, as it did not account for the relative proportion of NK cells in the GADEKILL, which ranged from 64.1% to 78.2% (Morandi et al. in press). Therefore, the comparable or higher NK cell cytotoxicity observed may suggest a greater antitumor activity of GADEKILL NK cells compared to purified NK cells, whereas γδ T lymphocytes conceivably contribute to the induction of target cell lysis.

Taking into account that the effector and cytotoxic functions of these immune cells arise from a balance of complex interactions among multiple receptors and ligands expressed on effector and target cells, we investigated whether NB cells are equipped with NCR ligands that bind activating receptors expressed on both γδ T and NK cells, as well as BTN that selectively activate γδ T lymphocytes. We observed that HA-TAG and calreticulin (i.e., NKp30 ligands), B7H6 (i.e., NKp46 ligand), BTN2A1, and BTN3A isoforms were consistently present on NB cell lines and patient-derived 3D tumor spheres. However, only B7H6 and BTN2A1 expression correlated with induced target cell lysis, supporting the concept that γδ T and NK cells cooperate to eliminate NB cells through parallel cytotoxic mechanisms. Of note, these two ligands may be used as a specific tumor target to enhance the antitumor activities exerted by NK and γδ T cells. In this regard, it has been reported that B7H6 is expressed on the cell surface of many tumor cell lines of different origins but not on hematopoietic cells from healthy individuals (75). This characteristic may render B7H6 a useful target for novel immunotherapies, as its engagement may eradicate chemoresistant solid tumors (76, 77). Furthermore, BTN members are expressed in various solid tumors (44), and, in the context of acute myeloid leukemia, decreased expression of BTN3A on the surface of blasts has been associated with poor survival (51). Nonetheless, the role of these molecules in neuroblastoma remains unexplored.

Taken together, our results justify further investigations on the GADEKILL in a formal phase I/II clinical trial in R/R HR-NB, also taking into account the well-known beneficial effects and safety of mature γδ T lymphocytes and NK cells infused with the graft in haploidentical HSCT. This therapeutic approach may be explored particularly in R/R HR-NB not expressing GD2, which currently remains without effective therapeutic options, through the infusion of donor-derived immune effector cells administered before T-cell-depleted haploidentical HSCT to achieve improved disease control before transplantation and/or administered afterward as enhanced donor lymphocyte infusions, with or without anti-GD2 mAb. In this regard, γδ T cells have been shown to represent the primary antitumoral T-cell population in pediatric neuroblastoma (78) and to induce tumor regression in preclinical models (70). Finally, the highest therapeutic efficacy of the GADEKILL may be anticipated in NB cases testing positive for BTN2A1 and B7H6.

## Data Availability

The raw data supporting the conclusions of this article will be made available by the authors, without undue reservation.
