# Supplementary figures and images for: The gamma delta T/NK cell product GADEKILL as a novel immunotherapeutic tool for neuroblastoma patients: role of B7H6 and BTN2A1 in tumor cell killing
Source: Front Immunol. 2026 Jan 30;17:1755500. doi: 10.3389/fimmu.2026.1755500 (PMC12901508; doi:10.3389/fimmu.2026.1755500)

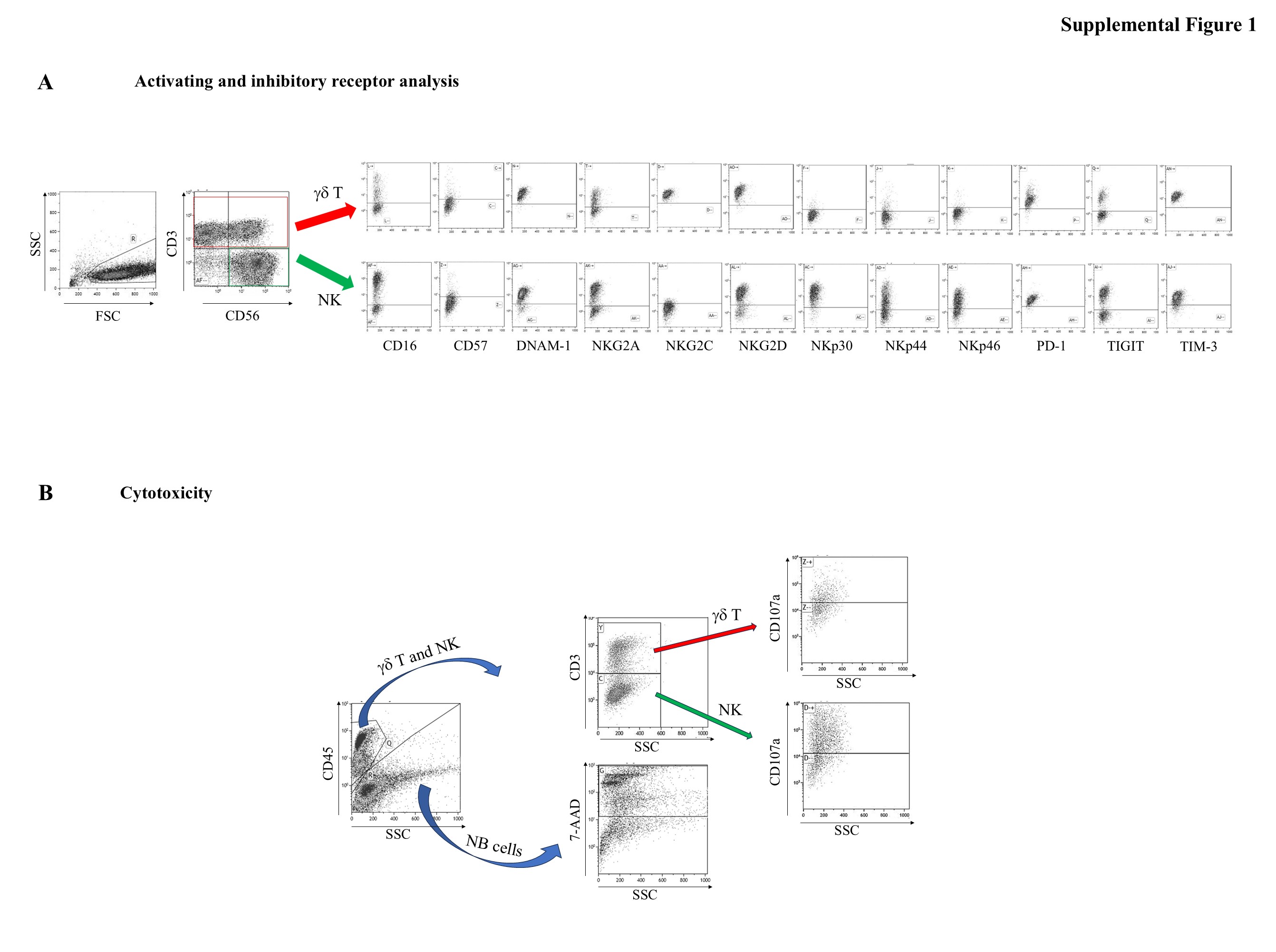

Supplement: Supplementary file 1 [file Image1.jpeg]

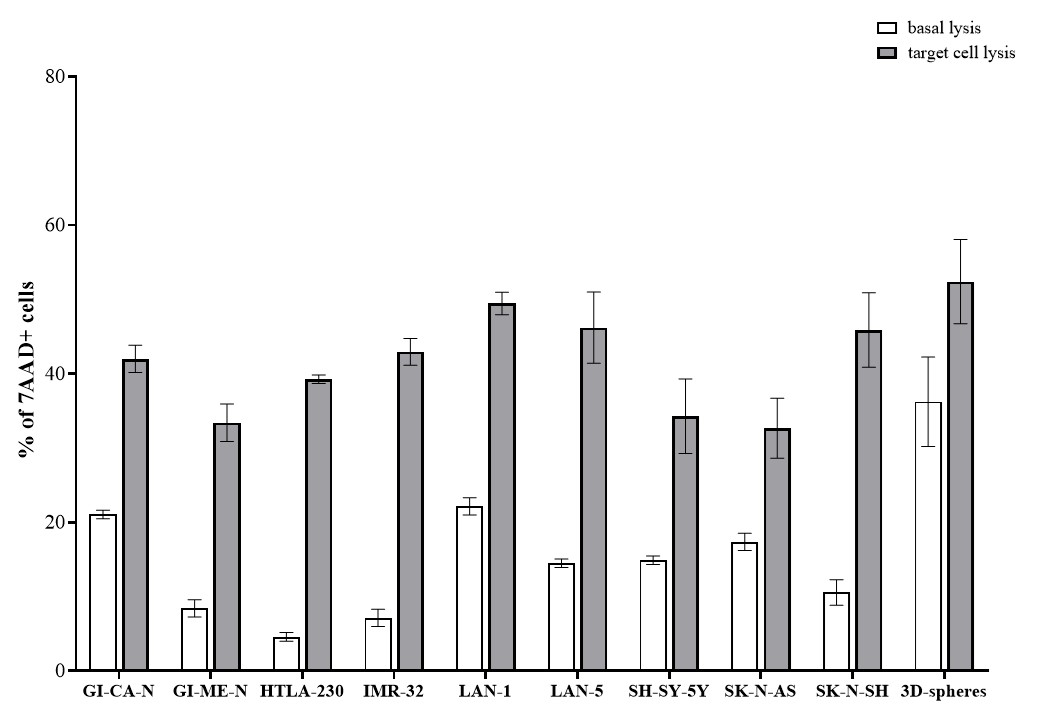

Supplement: Supplementary file 2 [file Image2.jpeg]
