# Supplementary material for: The gamma delta T/NK cell product GADEKILL as a novel immunotherapeutic tool for neuroblastoma patients: role of B7H6 and BTN2A1 in tumor cell killing
Source: Front Immunol. 2026 Jan 30;17:1755500. doi: 10.3389/fimmu.2026.1755500 (PMC12901508; doi:10.3389/fimmu.2026.1755500)
Supplement: Supplementary file 3 [file DataSheet1.pdf]

Supplemental Figure 1. Gating strategies. Panel A shows the gating strategy performed to analyze different surface receptors on $CD3^+$ $\gamma\delta$ T lymphocytes and $CD3^-CD56^+$ NK cells. **Panel B** shows the gating strategy used to discriminate $CD45^+$ cytotoxic effector cells and $CD45^-$ NB target cells. CD107a was analyzed in $CD3^+$ $\gamma\delta$ T lymphocytes or $CD3^-$ NK cells, whereas 7AAD was analyzed on target cells.

Supplemental Figure 2. Target cell lysis. Cell lysis was evaluated in cytotoxicity assay on NB target cells in the presence (grey bars) or absence (white bars) of effector cells. Three different batches of GADEKILL were used. Results are expressed as mean percentage of $7AAD^+$ cells (gated in $CD45^-$ cells) ± SD.
